# Diffusion tensor imaging for the study of early renal dysfunction in patients affected by bardet-biedl syndrome

**DOI:** 10.1038/s41598-021-00394-4

**Published:** 2021-10-21

**Authors:** Pasquale Borrelli, Miriam Zacchia, Carlo Cavaliere, Luca Basso, Marco Salvatore, Giovambattista Capasso, Marco Aiello

**Affiliations:** 1 IRCCS SDN, Via Emanuele Gianturco 113, 80131 Naples, Italy; 2 Department of Medical and Translational Sciences, University of Campania L. Vanvitelli, Naples, Italy; 3 Biogem, Research Institute for Molecular Biology and Genetics, Ariano Irpino, Italy

**Keywords:** Chronic kidney disease, Chronic kidney disease

## Abstract

Kidney structural abnormalities are common features of Bardet-Biedl syndrome (BBS) patients that lead to a progressive decline in renal function. Magnetic resonance diffusion tensor imaging (DTI) provides useful information on renal microstructures but it has not been applied to these patients. This study investigated using DTI to detect renal abnormalities in BBS patients with no overt renal dysfunction. Ten BBS subjects with estimated glomerular filtration rates over 60 ml/min/1.73m^2^ and 14 individuals matched for age, gender, body mass index and renal function were subjected to high-field DTI. Fractional anisotropy (FA), and mean, radial and axial diffusivity were evaluated from renal cortex and medulla. Moreover, the corticomedullary differentiation of each DTI parameter was compared between groups. Only cortical FA statistically differed between BBS patients and controls (p = 0.033), but all the medullary DTI parameters discriminated between the two groups with lower FA (p < 0.001) and axial diffusivity (p = 0.021) and higher mean diffusivity (p = 0.043) and radial diffusivity (p < 0.001) in BBS patients compared with controls. Corticomedullary differentiation values were significantly reduced in BBS patients. Thus, DTI is a valuable tool for investigating microstructural alterations in renal disorders when kidney functionality is preserved.

## Introduction

Bardet-Biedl syndrome (BBS) is a rare inherited disorder characterised by the dysfunction of several organs. To date, at least 24 genes (*BBS 1–24)*, most encoding proteins localised within the basal body of the primary cilium^1^, have been associated with human BBS. BBS patients are prone to develop renal failure^2^. Abnormal renal and urinary tract structures have been reported^3,4^; accordingly, chronic kidney disease (CKD) is considered the most common cause of morbidity and mortality. The renal phenotypes of both patients and mouse models are variable, although a tubulointerstitial defect is postulated to be a common substrate^5^ because of the low incidence of proteinuria and the high rate of urine concentration defects^6,7^.

In the last decade, magnetic resonance imaging (MRI) has been increasingly used for in vivo kidney studies. In addition to the clinical utility of MRI examinations for the assessment of several structural kidney abnormalities (e.g., autosomal dominant polycystic kidney disease^8^ and renal cancer^9,10^), recent studies have focused on the ability of kidney MRI to provide quantitative functional biomarkers for both diagnostic and prognostic purposes^11–16^. In this field, diffusion tensor imaging (DTI) provides useful insights into in vivo kidney analyses by measuring, noninvasively, physical properties related to kidney tissue microstructural integrity^17^. Indeed, the water movement analysis provided with the DTI allows the measurement of quantitative parameters, such as mean diffusivity (MD) and fractional anisotropy (FA), which reflect relevant features strictly related to the functional integrity of renal structures, such as tubules, collecting ducts and vessels^18,19^. In addition to the characterisation of healthy kidneys^20–22^, DTI has been successfully applied to detect renal damage in different kidney disorders, such as CKD^23–25^, kidney transplant^26–28^, diabetic nephropathy^29–31^, renal clear cell carcinoma^32^ and glomerulonephritis^33^. In these contexts, the absence of a contrast media makes the DTI analysis particularly suitable for subjects with kidney failure in which contrast media are highly contraindicated.

The principal aim of the present study was to investigate how microstructural parameters derived from DTI can be used as markers of kidney impairment in patients with BBS. Specifically, we assessed the utility of DTI in detecting early kidney alterations in a subset of BBS individuals with preserved renal function. To date, no dedicated quantitative MRI kidney studies in BBS are present in the literature. Indeed, only structural macro-abnormalities (e.g. kidney volume, cysts, malformations and fetal lobulations) have been detected by the application of MRI in BBS^34,35^. To the best of our knowledge, this is the first study focused on using DTI for in vivo studies on renal microstructural anomalies in BBS subjects. To achieve the present aim, DTI parameters of BBS subjects were compared with those of controls, which were matched for age, gender and renal function. Moreover, corticomedullary differentiation (CMD) obtained from DTI parameters was measured in patients and controls, and the right and left kidney were analysed separately using DTI parameters to assess possible asymmetrical alterations.

## Materials and methods

### Study participants

This study was approved by the local ethics committee (IRCCS Pascale) with protocol number 02/17. All the procedures adopted in this study were performed in accordance with the approved guidelines and regulations. Written informed consent was obtained from all the subjects enrolled in this study. From May 2018 to August 2019, 10 BBS patients referred to the Nephrology Unit of the University of Campania L. Vanvitelli were enrolled in the study. The diagnosis was based on Beales clinical criteria^36^. Retinal degeneration was assessed by ophthalmologic evaluation and learning disabilities were defined as defects in writing, reading and spelling, as well as deficiencies in memory, coordination and emotional maturity. Obesity was defined as a body mass index (BMI) higher than 30 kg/m^2^. Non-hypertensive subjects were defined as people with blood pressure < 140/90 mmHg in the absence of dedicated treatment. Non-diabetic individuals were defined as those with plasma HbA1c level < 7%, without a personal history of diabetes mellitus. Genetic validation was available for all the enrolled BBS patients. Renal function was assessed by estimating the glomerular filtration rate using the Chronic Kidney Disease Epidemiology Collaboration (CKD-EPI) equation with standardized plasma creatinine measurements. Genetic validation was available for all the enrolled patients.

The inclusion criteria were as follows: more than 18 years old, no contraindications to MRI examinations (i.e., metal implants, claustrophobia or pregnancy status), estimated glomerular filtration rate (eGFR) greater than 60 ml/min/1.73 m^2^.

In total, 14 subjects with no history of renal diseases, diabetes or hypertension were enrolled as the control group. The inclusion criteria of the controls were as follows: no contraindications to MRI examinations, eGRF greater than 60 ml/min/1.73m^2^, absence of renal pathologies, diabetes, hypertension or metabolic abnormalities. Moreover, control subjects were selected by matching the gender, age range and BMI of the BBS group members.

### MRI protocol

Both BBS and control subjects underwent MRI examinations with the same acquisition protocol using a 3 T scanner (Biograph mMR; Siemens, Erlangen, Germany), equipped with a 4-channel phased-array body coil.

Anatomical T1- and T2-weighted images, covering both the right and left kidney, were acquired to examine kidney morphology. The T1-weighted images were acquired with the 2D Fast Low Angle Shot (FLASH) sequence, whereas T2-weighted images were obtained with the half-Fourier acquisition single-shot turbo spin echo (HASTE) sequence. Both FLASH and HASTE images were acquired with a coronal orientation in breath-hold.

For DTI images, a fat-saturated twice-refocused EPI sequence was performed with a respiratory triggering, 500 s/mm^2^ as diffusion weighting and three averages each of which with six different diffusion directions at b = 500 s/mm^2^ and two volumes at b = 0 s/mm^2^. A diffusion weighting of b = 500 s/mm^2^ was chosen as a good compromise between image quality and pure diffusion measurement (i.e., by neglecting the perfusion phenomena) and considering the mean diffusivity of the kidneys as reported in the literature^37–39^. Additional parameters of the EPI sequence were as follows: oblique-coronal orientation, repetition time 1800 ms/echo time 74 ms; 152 × 152 matrix; 319 × 319 mm^2^ field of view; slice thickness of 3 mm; 2.1 × 2.1 mm^2^ pixel size; 36 slices; 2 as the parallel imaging accelerator factor and left to right as the phase-encoding direction. Another set of DTI images was acquired with the same parameters by only blipping the phase-encoding direction (right to left) for the subsequent geometric distortion correction^39^. The total scan time varied from 15 to 20 min, depending on the individual’s respiratory cycle.

### Image analysis

Prior to image analyses, the images were de-identified and visually checked for image artefacts. In particular, the raw image data in the DICOM format were elaborated with the software provided with the scanner to remove the DICOM tags correlated with patient’ data, and then, they were transferred to a workstation for the subsequent image analyses.

The acquired images were analysed by an expert radiologist (C.C.) with more than 8 years of experience in abdominal imaging. In particular, the radiologist assessed the image quality by visually checking for the absence, or low presence, of image artefacts such as gross movement, ghosting and signal loss. Moreover, both the FLASH and HASTE images were inspected to detect renal structural abnormalities (i.e. cysts or fetal lobulations).

The DTI images were pre-processed for mitigating image artefacts such as movement, eddy-currents and geometric distortion. The pre-processing procedure was chosen to provide the highest test–retest reproducibility results, as reported in Borrelli et al.^39^. In particular, the movement between and within the DTI series and the distortions induced by eddy-currents were corrected using the *eddy* command, whereas the susceptibility-induced geometric distortions were corrected using the *topup* function. Both dedicated routines were implemented in the FMRIB-FSL library (v. 6.0.0)^40^. For the geometric distortion correction, an approach based on the estimation of the susceptibility off-resonance field^41^ was adopted using additional DTI data acquired with the opposite phase-encoding direction.

Parametric maps derived from the pre-processed DTI images were evaluated using MRtrix3 toolbox^42^. In particular, MD, FA, radial diffusivity (RD) and axial diffusivity (AD) were evaluated from the eigenvalues of the calculated diffusion tensor. Moreover, the mean image among b = 0 s/mm^2^ DTI images (denoted as b0) was calculated for further processing.

### Regions of interest (ROIs) definitions

**Figure 1:**
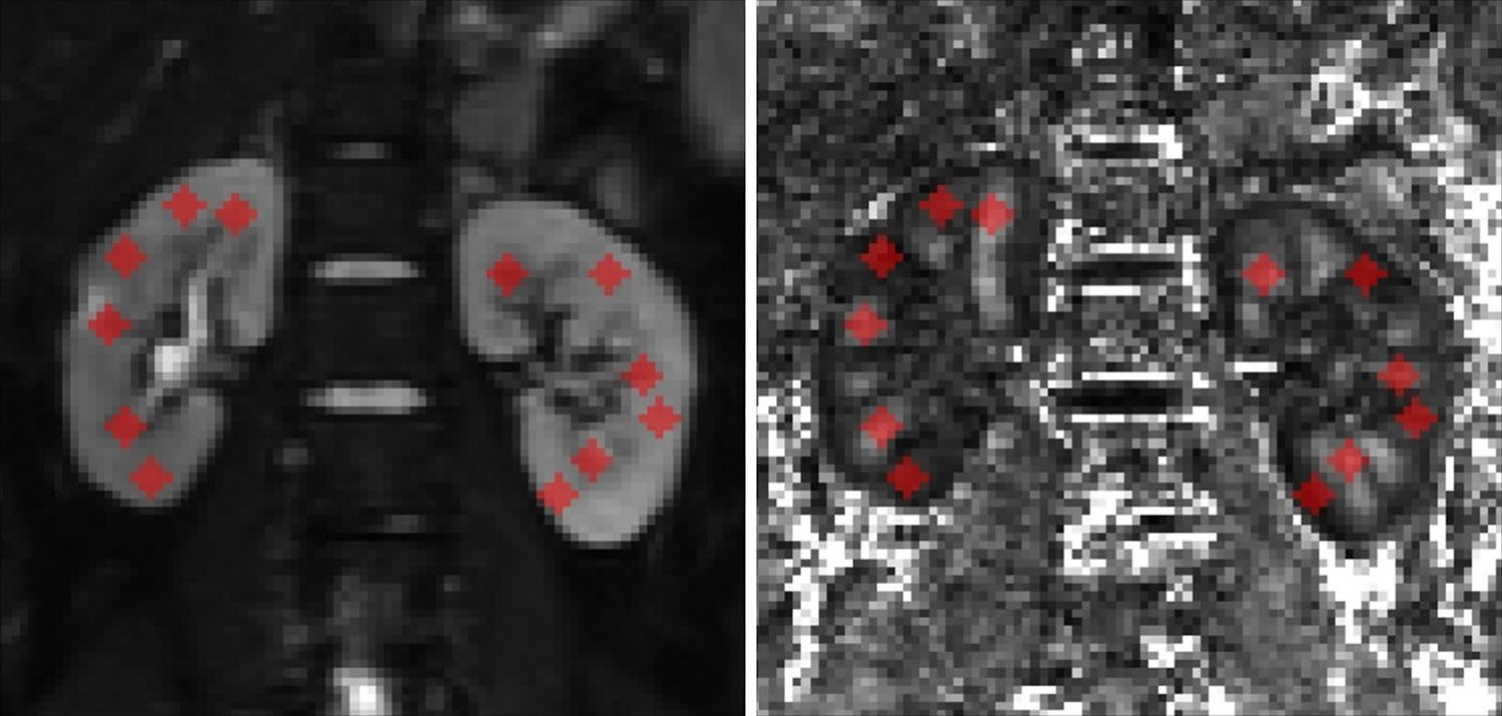
Kidneys regions of interest (ROIs) in a Bardet–Biedl syndrome patient. Baseline, b = 0 s/mm^2^ (left) and fractional anisotropy (right) of a BBS subject included in the present study overlaid with ROIs (red circles). The ROIs were positioned in the upper, middle and inferior kidney poles in both the medullary and cortical tissues.

ITK-SNAP (v. 3.6.0)^43^ was used to annotate ROIs. The ROI generation procedure consisted of manually drawing circular 58 mm^2^ ROIs at the upper, middle and inferior poles of both cortical and medullary kidney tissues. ROI selection was performed by the radiology technician (L.B.) and confirmed by the radiologist (C.C.). For each subject included in the present study, the ROIs were drawn on coronal slices in the central section of the kidney (i.e. close to the renal hilum) paying attention to avoid the renal pelvis, vessels, calyces and, eventually, renal cysts and fetal lobulations. ROI placement was performed on the b0 image and the corresponding FA map was used to confirm the accuracy of the annotation procedure. The purpose of constraining the ROI placement on a single well-identifiable slice was to create the most reproducible procedure for revealing abnormalities in a diffuse (i.e., non-focal) renal disease, such as BBS. Both right and left kidneys were annotated, resulting in 12 different ROIs for each subject (Fig. 1). Finally, the ROIs were copied to each parametric map.

### Statistical analyses

For the following statistical analysis, both cortical and medullary means and standard deviations (SD) from each DTI map were calculated by averaging the ROI values from the upper, middle and inferior poles of each kidney. Moreover, the obtained values were averaged for the left and right kidneys, thereby obtaining a single measurement for both kidneys.

In addition, the CMD derived from each DTI parameter was calculated as the ratio between cortical and medullary mean values. A value close to 1 reflected poor CMD, whereas values farther from 1 represented good differentiation between renal cortical and medullary tissues. By incorporating DTI values evaluated from healthy subjects^18,19,21,44^, FA and AD maps should result in CMD values less than 1 owing to higher FA and AD values in the medulla compared with the renal cortex. Conversely, because MD and RD values reflect the mean diffusivity and the diffusivity parallel to the principal diffusion direction respectively, the CMD of a healthy kidney should result in a value greater than 1.

The statistical analysis was performed using MATLAB r2018a (Mathworks, Inc.).

To investigate renal microstructural injuries in BBS subjects, three specific statistical analyses were performed:

- DTI parameters: for each parametric map, the Wilcoxon rank sum test was performed between mean values of both kidneys, separately for cortical and medullary values, from BBS and control subjects to compare between the two groups.
- CMD analysis: the Wilcoxon rank sum test was applied to CMD values to investigate CMD differences between BBS subjects and controls.
- Kidney asymmetry: the statistical difference between measurements from right and left kidneys within both BBS and control subjects was assessed using the Wilcoxon rank sum test to determine whether DTI parameters of the left kidney significantly differed from those of the right kidney.

For all the statistical tests, p < 0.05 was considered statistically significant.

## Results

### Patients cohort

The details of patients and controls are reported in Table 1. The mean ages were 33 ± 4 and 28 ± 8 years, respectively, and the eGFRs were in the normal range for both patients and controls, with mean values of 103.90 ± 19.34 and 95.05 ± 16.01 ml/min/1.73 m^2^, respectively. Additional clinical features of BBS patients included retinal dystrophy in 9/10 patients and learning disabilities were detected in 6 out of 10 BBS individuals. The mean BMI was of 26.96 kg/m^2^ in BBS individuals, which was not significantly different from that of the controls. Genetic analysis was available for all the BBS patients, and after a next generation sequencing analysis, 3/10 patients showed no mutations in known BBS-related genes. The remaining patients were homozygous for the *BBS4* (n = 2), *BBS12* (n = 2), *BBS1,10* and *9* (n = 1, each) variants. None of the controls had signs of functional and/or structural renal anomalies.

**Table 1:** Main features of Bardet–Biedl syndrome (BBS) patients and controls.

Parameter	BBS	Controls	*p *Value
Age (mean ± SD)	28 ± 8 years	33 ± 4 years	0.982
Gender (female/male)	5 / 5	4 / 10	0.285
BMI (mean ± SD)	26.96 ± 4.78	24.82 ± 1.48	0.111
eGFR (mean ± SD)	103.90 ± 19.34	95.05 ± 16.01	0.292

BMI: body mass index (Kg/m^2^); eGFR: estimated glomerular filtration rate (ml/min/1.73m^2^); SD: standard deviation.

### MRI examinations

**Figure 2:**
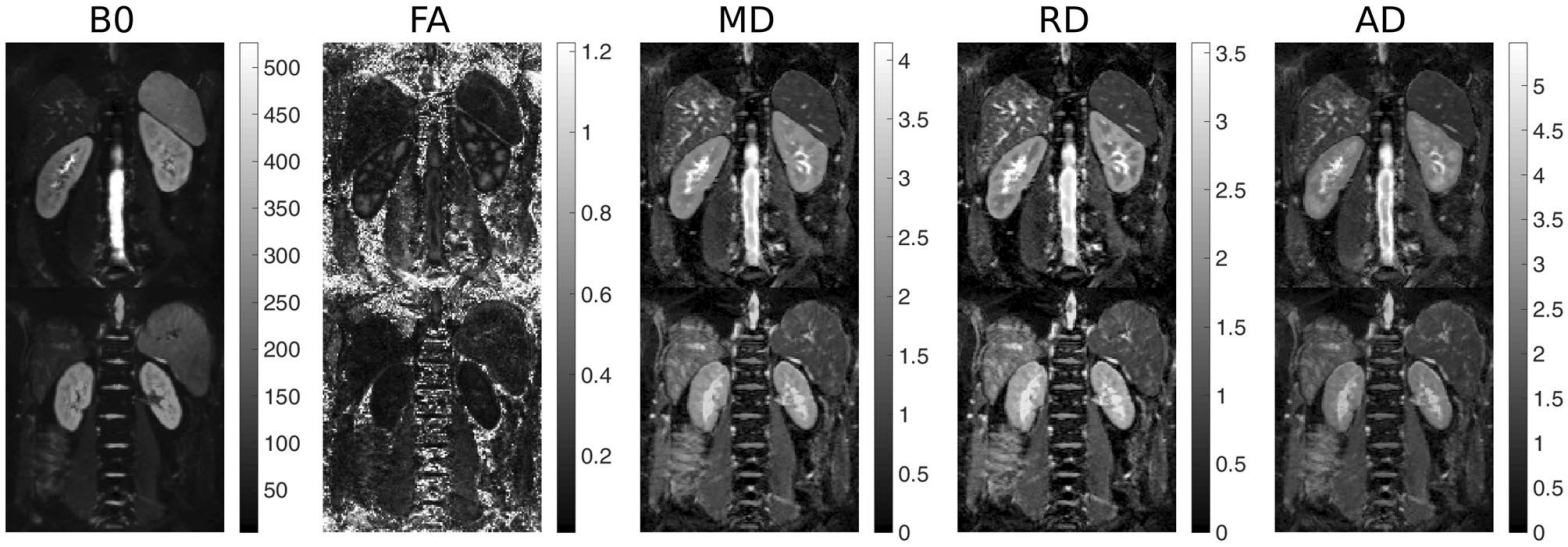
Diffusion Tensor Imaging maps for a Bardet–Biedl syndrome and a control subject. The baseline, b = 0 s/mm^2^ DTI image (B0, left) and derived parametric maps (FA: fractional anisotropy, MD: mean diffusivity, RD: radial diffusivity and AD: axial diffusivity, respectively from left to right) for a control (upper) and BBS subject (lower). The FA images are represented in arbitrary units, whereas the MD, RD and AD images are represented in 10^–3^ mm^2^/s.

After the preliminary visual assessment of image quality, all the acquired images, both from BBS and controls, were suitable for the image analyses procedures. An example of DTI maps, with related CMD values, for a BBS and a control subject included in the present study is shown in Fig. 2.

**Figure 3:**
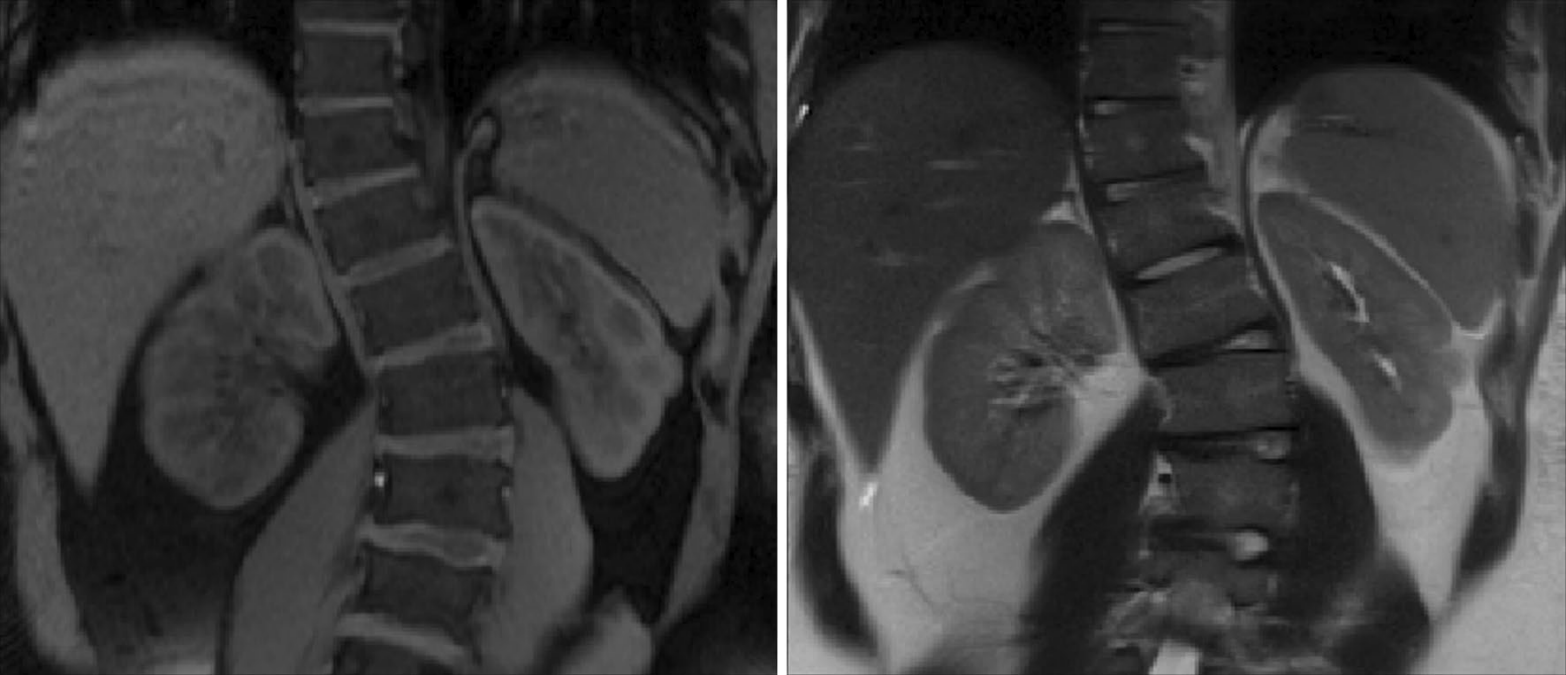
T1- (left) and T2-weighted (right) MRI images of a BBS subject included in the present study. The morphological analysis shows fetal lobulations at the inferior pole of the left kidney.

Renal structural abnormalities were found in six BBS subjects. In particular, three BBS subjects presented renal fetal lobulations (Fig. 3) and four BBS subjects exhibited cysts in the cortical renal tissue with maximum diameter < 6 mm. No morphological kidney abnormalities were found in control subjects.

### DTI parameters

**Figure 4:**
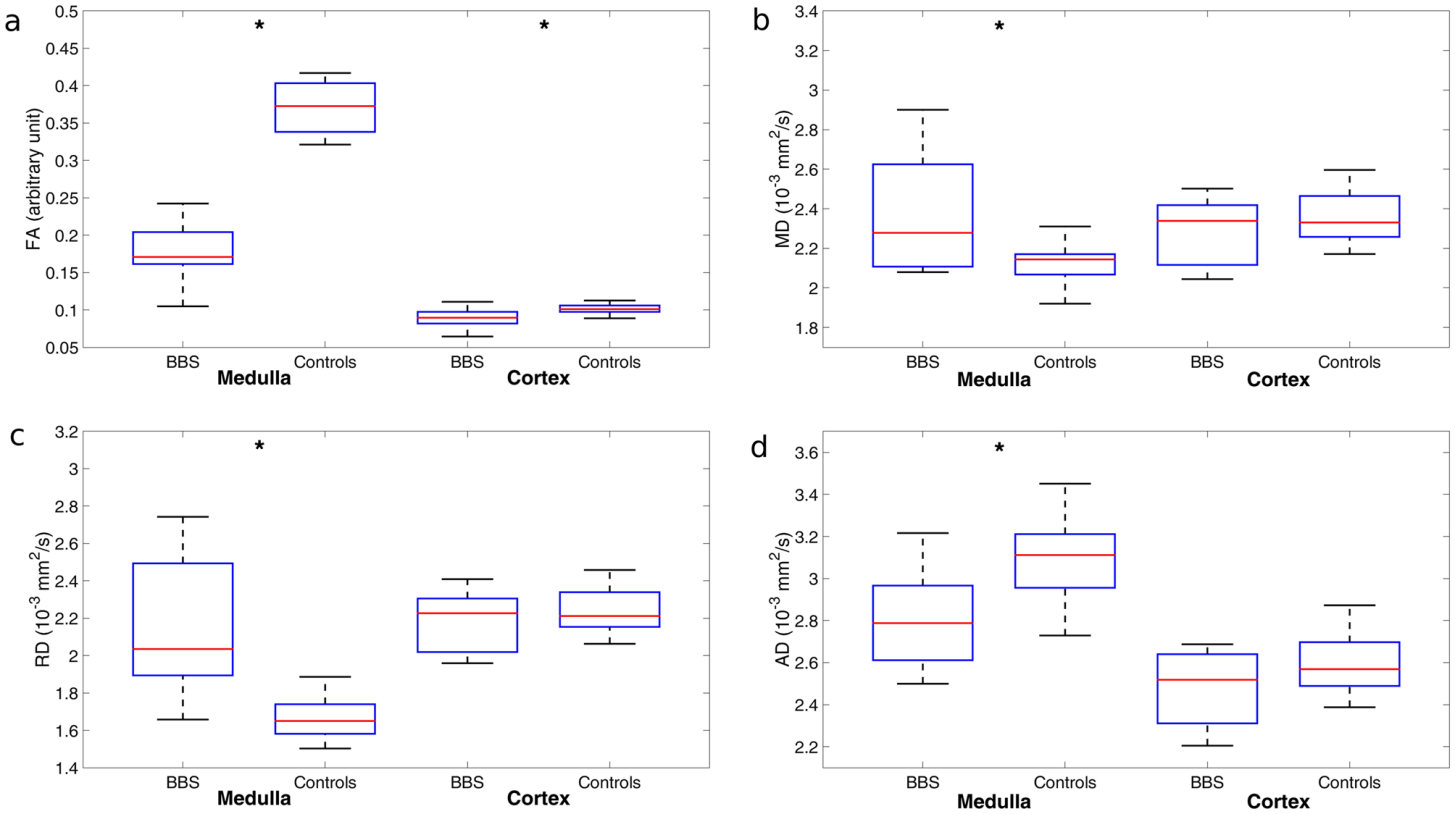
Distribution of both medullary and cortical mean values arising from fractional anisotropy (**a**), mean diffusivity (**b**), radial diffusivity (**c**) and axial diffusivity (**d**) for BBS and control subjects. Red lines correspond to median values and the blue boxes include from the 25th to 75th percentile of the distribution. The asterisks indicate statistically significant differences between BBS subjects and controls.

The distributions of the medullary and cortical values for both BBS and control subjects are shown in Fig. 4 and the means and SD values from BBS and controls subjects are reported in Table 2. All the DTI parameters from the renal medulla were statistically different between the two groups (Table 2), whereas only cortical FA values differentiated BBS from controls (p = 0.016). In particular, medullary FA and AD values were higher in controls than in BBS patients, whereas medullary MD and RD values were higher in BBS subjects than in controls (Table 2).

**Table 2:** Bardet–Biedl syndrome (BBS) and controls median and interquartile ranges (in square brackets) for DTI parameters from both the cortical and medullary regions.

	BBS	Controls	*p *Value
Cortical FA	0.089 [0.014]	0.101 [0.008]	**0.033**
Medullary FA	0.171 [0.034]	0.373 [0.058]	** < 0.001**
Cortical MD	2.339 [0.271]	2.330 [0.190]	0.429
Medullary MD	2.278 [0.415]	2.144 [0.095]	**0.043**
Cortical RD	2.227 [0.262]	2.212 [0.171]	0.501
Medullary RD	2.035 [0.474]	1.650 [0.140]	** < 0.001**
Cortical AD	2.518 [0.308]	2.568 [0.197]	0.151
Medullary AD	2.788 [0.329]	3.111 [0.236]	**0.021**

FA: fractional anisotropy; MD: mean diffusivity; RD: radial diffusivity; AD: axial diffusivity.

FA values are expressed in arbitrary units, whereas MD, RD and AD values are reported in 10^–3^ mm^2^/s. p-values < 0.05 are represented in bold.

### CMD

The details of the CMD values derived from each DTI parameter for both BBS and control subjects are shown in Table 3. All the CMD values, with the exception of AD, substantially differed between BBS and controls, with the CMD values being higher in controls than BBS subjects. Notably, the mean CMD derived from MD resulted in an inverted behaviour between BBS (CMD < 1) and controls (CMD > 1).

**Table 3:** Corticomedullary differentiation, reported as median and interquartile ranges (in square brackets), which were calculated as the ratios between cortical and medullary mean values for each DTI parameter.

	BBS	Controls	p-value
FA	0.546 [0.141]	0.268 [0.045]	** < 0.001**
MD	0.956 [0.053]	1.100 [0.063]	** < 0.001**
RD	0.992 [0.080]	1.350 [0.072]	** < 0.001**
AD	0.889 [0.074]	0.828 [0.063]	0.063

FA: fractional anisotropy; MD: mean diffusivity; RD: radial diffusivity; AD: axial diffusivity; BBS: Bardet-Biedl syndrome.

p-values < 0.05 are presented in bold.

### Kidney asymmetry

Neither BBS subjects nor controls showed statistical differences in DTI parameters from the left and right kidney (Table S1). Moreover, as shown in Table S1, the differences in DTI parameters between groups remained significantly different between patients and controls when the analysis was separately performed for each kidney.

## Discussion

This is the first study using DTI to characterise renal abnormalities in BBS patients before the onset of clinically evident renal dysfunction. The latter is an important cause of morbidity and mortality in BBS patients and, to date, the exact pathomechanisms leading to kidney disease in these patients is unknown. Moreover, because CKD in BBS, as well as in the general population, is a progressive disorder characterised by a time-dependent progression, the early diagnosis of kidney abnormalities will have great clinical impact, because it allows the determination of patients at risk of progressing renal disease.

To date, limited information is available in the literature on markers of early CKD in BBS patients. As in non-syndromic individuals, an increased plasma creatinine levels is not an early index of kidney dysfunction. This study demonstrates that the major parameters used to address renal dysfunction are sometimes not sufficient to detect small kidney defects that may predispose a patient to CKD progression^31^. For this purpose, we selected BBS patients with eGFR values greater than 60 ml/min/1.73 m^2^. This subgroup of individuals included young adult subjects with plasma creatinine level in the normal range. Moreover, consistent with the well-documented theory that kidney disease in this setting is not primarily a glomerular disorder, these patients did not show significant increases in urine protein excretion. Thus, the BBS patients we selected did not show alterations in the major parameters used in the clinical setting to address renal dysfunction.

As a primary finding, DTI was able to discriminate BBS patients from age-gender-eGFR-BMI matched controls. Indeed, DTI values differed between BBS and controls, especially in the renal medulla, in which more tubular structures are represented. The altered values in the medullary renal tissues of BBS subjects may reflect the compromised integrity of collecting ducts as a sign of early tubulointerstitial injuries, which is a common feature that causes urine concentration dysfunction in BBS^45^. Among the characteristics, the FA was able to best discriminate BBS from controls because it measures the preferential directionality of water molecules within tissues. In this setting, alterations in FA values may reflect microstructural changes in the kidneys of BBS subjects. Our findings are in-line with a recent study in which FA values identified early renal damage in diabetic patients with normal BMI and eGFR values^31^.

Moreover, poorer CMD was revealed in BBS patients, indicating that the CMD may represent an additional index of early renal abnormalities in BBS. The reduction of CMD in BBS patients was likely owing to the significant alterations in the DTI values of medullary tissues compared with the controls. Therefore, the kidney injuries measured using DTI were not specifically related to the left or right kidney, which confirmed the symmetry of CKD-related renal damage in BBS patients^3^.

The primary limitation of the present study is the small sample size. However, the limited number of subjects is due to the rarity of BBS and the presence of constraints on MRI examinations (i.e., BMI). As a second limitation, no direct comparison of the presented findings were performed with DTI parameters obtained from subjects with other kidney structural abnormalities (such as congenital renal dysplasia). In addition, because the morbidly obese cannot undergo MRI studies, the analysis was applied to mainly overweight BBS patients with no severe obesity. In this scenario, obesity may confound the assessment of kidney diffusion parameters based on the DTI^46^. Finally, the genetic variability in the BBS subjects, together with the small sample size, made it difficult to assess genotype to phenotype correlations. Further studies with larger numbers of BBS subjects may untangle possible associations between specific BBS gene mutations and kidney phenotypes using the DTI technique.

It is worth underlining the importance of the non-invasiveness of renal DTI. Indeed, as specified in the methodological section, the DTI technique does not require the use of any contrast media and, therefore, is suitable for renal pathologies, thus deserving attention for the translation in clinical practice.

In conclusion, this study confirmed the ability of the DTI technique to reveal renal abnormalities before the clinical onset of renal dysfunction, demonstrating its suitability as a tool for detecting early renal injuries.

## Supplementary Information

Supplementary Information.

## Data Availability

MR data can be made available under request to the corresponding author (pending the approval of the Research Ethics Board).
