# Supplementary material for: Diffusion tensor imaging for the study of early renal dysfunction in patients affected by bardet-biedl syndrome
Source: Sci Rep. 2021 Oct 21;11:20855. doi: 10.1038/s41598-021-00394-4 (PMC8531379; doi:10.1038/s41598-021-00394-4)
Supplement: Supplementary file 1 — Supplementary Information. [file 41598_2021_394_MOESM1_ESM.pdf]

Diffusion Tensor Imaging for the study of early renal dysfunction in patients affected by Bardet-Biedl Syndrome

Supplementary Information

Pasquale Borrelli, Miriam Zacchia, Carlo Cavaliere, Luca Basso, Marco Salvatore, Giovambattista Capasso and Marco Aiello

Supplementary Information

Table S1. Kidney asymmetry. BBS and controls median and interquartile range (in square brackets) of DTI parameters for both cortical and medullary regions separately evaluated for left and right kidney. FA values are expressed in arbitrary units, while MD, RD and AD values are reported in 10^{-3} mm^2/s. p-values < 0.05 are represented in bold format.

			BBS	Controls	p-value
FA	Cortical	Right	0.086 [0.023]	0.102 [0.012]	0.095
		Left	0.089 [0.018]	0.099 [0.008]	0.065
		p-value	0.970	0.395	
	Medullary	Right	0.168 [0.060]	0.383 [0.058]	**<0.001**
		Left	0.177 [0.047]	0.368 [0.034]	**<0.001**
		p-value	0.734	0.629	
MD	Cortical	Right	2.289 [0.245]	2.275 [0.217]	0.747
		Left	2.300 [0.260]	2.363 [0.093]	0.253
		p-value	0.910	0.135	
	Medullary	Right	2.282 [0.453]	2.153 [0.164]	0.074
		Left	2.266 [0.259]	2.125 [0.167]	0.057
		p-value	0.791	0.801	
RD	Cortical	Right	2.188 [0.228]	2.146 [0.190]	0.977
		Left	2.200 [0.264]	2.235 [0.104]	0.305
		p-value	0.733	0.113	
	Medullary	Right	2.013 [0.521]	1.658 [0.123]	**0.001**
		Left	2.046 [0.276]	1.643 [0.130]	**<0.001**
		p-value	0.998	0.836	

AD	Cortical	Right	2.459 [0.260]	2.535 [0.255]	0.429
		Left	2.499 [0.239]	2.600 [0.097]	0.121
		p-value	0.791	0.175	
	Medullary	Right	2.813 [0.387]	3.068 [0.349]	0.074
		Left	2.741 [0.351]	3.119 [0.244]	**0.004**
		p-value	0.910	0.945	

FA: fractional anisotropy; MD: mean diffusivity; RD: radial diffusivity; AD: axial diffusivity; BBS: Bardet-Biedl syndrome.
